# Mögliches Gadolinium-Depositionssyndrom – ein Fallbericht

**DOI:** 10.1055/a-2661-4679

**Published:** 2025-08-04

**Authors:** Torsten Diekhoff, Wolfgang Böhmerle, Jan-Piet Habbel

**Affiliations:** 1 Department of Radiology 477107 Brandenburg Medical School Theodor FontaneRüdersdorfBBGermany; 2 Department of Neurology 14903 Charité Universitätsmedizin BerlinBerlinGermany; 3 Department of Hematooncology 14903 Charité Universitätsmedizin BerlinBerlinGermany

**Keywords:** gadolinium, MR-imaging, abdomen

## Einleitung

Durch die Ehefrau des Schauspieler Chuck Norris publik gemacht, steht das sogenannte Gadolinium-Depositionssyndrom (Gadolinium Deposition Disease, GDD) seit der Erstbeschreibung im Jahr 2016 1 im Zentrum kontroverser Diskussionen und beschreibt eine Erkrankung, bei der Patienten trotz normaler Nierenfunktion nach Gabe gadoliniumhaltiger Kontrastmittel (GBCA) teils persistierende Symptome wie Fatigue, brennende Schmerzen der Extremitäten, Muskelfaszikulationen und Hautveränderungen entwickeln 2 . Pathophysiologisch vermutet man eine chronische oder schubartige Entzündungsreaktion durch im Gewebe retinierte Gadolinium-Ionen, insbesondere nach Verwendung linearer GBCA 3 . Zwar ist die Ablagerung von Gadolinium in Geweben wie Gehirn, Haut oder Knochen belegt, doch die klinische Relevanz bleibt fraglich und ist weiterhin Gegenstand intensiver Forschung. Besonders selten wurden bislang Fälle nach Verabreichung leberspezifischer linearer GBCA wie Gadoxetat (Primovist) beschrieben. Der vorliegende Fall schildert daher einen ungewöhnlichen Verlauf wiederholter Gadoxetat-Gaben und trägt zur laufenden Debatte um Existenz und klinische Bedeutung des GDD bei.

## Fallbeschreibung

Wir schildern den Fall einer 32-jährigen Patientin, die sich zur Abklärung eines Mamma-Tastbefundes vorstellte. Als Vorerkrankungen waren eine operativ versorgte zervikale Bandscheibenextrusion und ein Heuschnupfen bekannt. Der Gesamtverlauf ist tabellarisch in Tab. 1 aufgelistet.

**Table:** **Tab. 1** Zeitliche Auflistung des onkologischen Verlaufs, der Kontrastmittelmengen und der Symptome, die auf GDD zurückgeführt werden. M1: Gadotersäure, M2: Gadobutrol.

Q	Jahr	Onkologie	Gadoxetat	M1	M2	Symptome
3	1	Einleitung Systemtherapie mit Paclitaxel, Trastuzumab / Pertuzumab und Denusomab	10,0ml			Leichte Polyneuropathie der Finger
4			10,0ml	16ml		
1	2	Umstellung auf Erhaltungstherapie mit Trastuzumab, Pertuzumab, Denusomab und Tamoxifen	10,0ml			Nächtliche Unruhe, krampfartige Muskelschmerzen in der Wade
2			10,0ml			
3			10,0ml			
4			10,0ml			
1	3					
2			10,0ml			
3						Etwas Besserung der Beschwerden
4			10,0ml			
1	4					
2			10,0ml			
3		Neue cerebrale Metastasen				Wieder zunehmende Beschwerden, brennende Schmerzen am Fußrücken, Berührungsempfindlichkeit
4		Tiefe Beinvenenthrombose	10,0ml	15ml	22,2ml	
1	5		10,0ml		7,7ml	
2		Rezidiv cerebrale Metastasen	10,0ml	32ml		
3		Therapiewechsel auf T-DM1	10,0ml	16ml	16,4ml	
4		Neurologische Vorstellung	10,0ml			Etwas Besserung unter Physiotherapie und Pregabalin
1	6	Weiter gutes Ansprechen auf T-DM1 mit bildgebender Remission	10,0ml		7,9ml	
2			7,5ml		15,7ml	
3			8,0ml		15,8ml	
4			8,0ml	16ml		Zeitlicher Zusammenhang zu MRT-Untersuchungen wird klar; Hautverfärbungen werden abgeklärt
1	7	Wechsel auf Capecitabine, Tucatinib, Trastuzumab bei cerebralem Progress	7,0ml	32ml		
2			8,0ml		7,4ml	
3		Umstellung auf T-DXd bei erneutem Progress				Deutliche Besserung der Symptome
4				32ml		Weitgehende Beschwerdefreiheit
1	8	Remission unter T-DXd			8,3ml	
2					8,3ml	
3					8,0ml	
4						

### Onkologische Aspekte

In der Initialdiagnostik ergab sich ein primär hepatisch und ossär metastasiertes Mammakarzinom mit einer Her2neu Überexpression und positiv für Östrogen- (100%) und Progesteronrezeptoren (90%). Es wurde eine Systemtherapie mit Paclitaxel, Trastuzumab und Pertuzumab analog der Cleopatra-Studie sowie eine Osteoprotektion mit Denosumab begonnen 4 . Nach gutem Ansprechen und bildgebender Komplettremission wurde 6 Monate nach Erstvorstellung auf eine Erhaltungstherapie mit Tamoxifen in Kombination mit den beiden Antikörpern Trastuzumab und Pertuzumab umgestellt.

Drei Jahre und einen Monat nach Erstvorstellung traten Hirnmetastasen auf, die operativ reseziert und nachbestrahlt wurden. Unter wiederholten Rezidiven wurde auf die Systemtherapie auf Trastuzumab-Emtansin (T-DM1), dann Capecitabine, Tucatinib und Trastuzumab sowie schließlich auf Trastuzumab-Deruxtecan (T-DXd) umgestellt, die bis zum Erscheinen des Artikels eine bildgebende Komplettremission erhält.

### Radiologische Aspekte

**Abb. 1:**
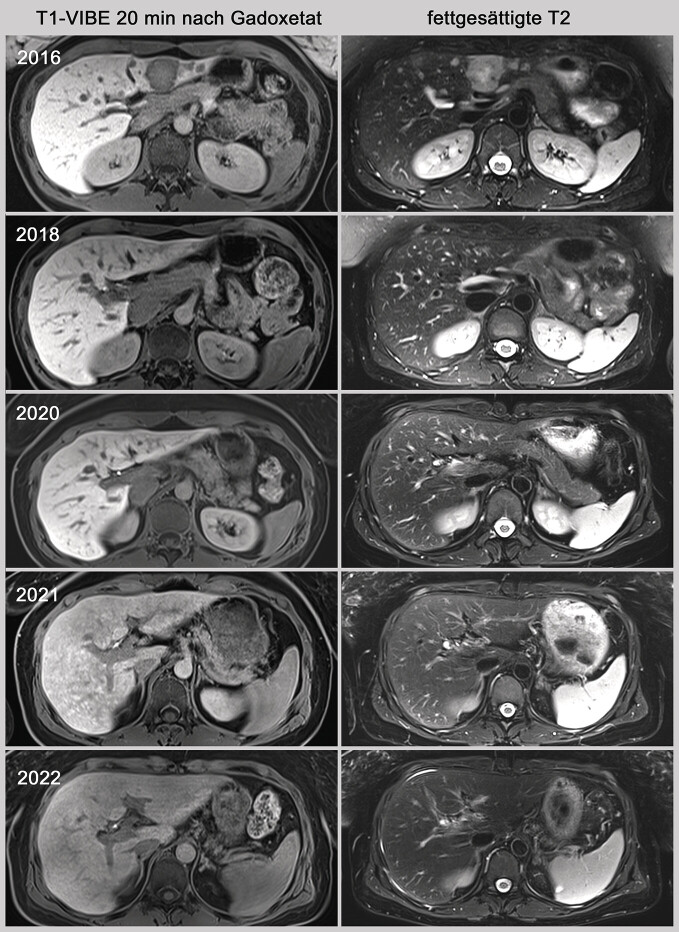
Verlauf der Bildgebung in der Leber-MRT. Nach dem Initialbefund eines primär hepatisch (und ossär, nicht abgebildet) metastasierten Mamma-Ca im Jahr 2016 zeigten die Verlaufs-MRTs in den Jahren 2018 und 2020 ein komplettes Ansprechen. Im Verlauf seit 2020 zeigt sich ein zügiger Verlust der hepatozellulären Aufnahme von Gadoxetat in den Jahren 2021 und 2022, sodass schließlich auf makrozyklische Kontrastmittel gewechselt wurde.

Es wurde sich initial für ein Stagingkonzept mit Gadoxetat-gestützter Leber-MRT in Kombination mit einer nativen Thorax-CT entschieden. Die Bildgebungsintervalle waren auf drei Monate ausgelegt; in Phasen guten Therapieansprechens wurden sie auf sechs Monate ausgedehnt. Im Verlauf konnte unter der T-DM1- und T-DXd-Therapie eine abnehmende Hepatozytenaufnahme von Gadoxetat beobachtet werden ( Abb. 1 ). Daher erfolgte im sechsten Jahr die Umstellung auf ein makrozyklisches Gadolinium-haltiges Kontrastmittel (Gadoterat bzw. Gadobutrol). Insgesamt waren in den sechs Jahren 40 MRT-Untersuchungen erfolgt, wobei 20 Untersuchungen mit einer kumulativen Gesamtmenge von 188,5 ml Gadoxetat (entsprechend 0,59 mmol/kg Körpergewicht), 8 Untersuchungen mit insgesamt 127 ml Gadotersäure (0,79 mmol/kg) und 12 Untersuchungen mit insgesamt 93,1 ml Gadobultrol (1,16 mmol/kg) durchgeführt wurden. Nach vollständigem Abklingen der neurologischen Symptomatik wurden in den folgenden drei Jahren 3 Untersuchungen mit insgesamt 49 ml Gadotersäure und 7 Untersuchungen mit insgesamt 58.6 ml Gadobutrol durchgeführt, ohne dass es zu neuerlichen Beschwerden kam.

### Symptome

Mit Einleitung der Paclitaxel Chemotherapie stellte sich eine geringgradige Polyneuropathie der Finger ein, die sich nach Umstellung auf die Erhaltungstherapie langsam besserte. Erstmals im Jahr nach Therapiebeginn beschreibt die Patientin nächtliche Unruhe ähnlich einem Restless-Legs-Syndrom. Es traten Muskelkrämpfe auf, die die Patientin selbst mit oralem Magnesium und Calcium therapierte. Anderthalb Jahre später nahmen die Beschwerden etwas ab, verschwanden aber nie vollständig. Insgesamt wurde die Symptomatik auf die anhaltende Therapie und Belastung zurückgeführt. Im vierten Therapiejahr nahmen die Beschwerden wieder zu und wurden durch brennende Schmerzen und Berührungsempfindlichkeit im Bereich beider Fußrücken ergänzt. Eine Ultraschall-Untersuchung im Rahmen der Umfelddiagnostik bei zerebralem Rezidiv entdeckte eine Unterschenkelvenenthrombose, die mit Antikoagulation behandelt wurde – eine Besserung der Beschwerden stellte sich unter dieser Behandlung nicht ein.

Im fünften Jahr erfolgte eine erste Zuweisung zur neurologischen Spezialsprechstunde bei exazerbierter schmerzhafter Dysästhesie beider Fußrücken und Zehen, welche die Patientin in einen zeitlichen Zusammenhang mit der letzten T-DXd-Gabe brachte.

Die neurologische Untersuchung ergab:

- Keine Störungen der Motorik oder Koordination, sicherer Stand
- Geringe Hypästhesie, Hypalgesie und Hypthermästhesie der Fingerspitzen und des Fußrückens

Insgesamt wurde der Verdacht auf ein geringes polyneuropathisches Schmerzsyndrom vom distal-symmetrischen Typ, ggf. bei Paclitaxel- und T-DM1-induzierter Polyneuropathie, gestellt. Mittels lokaler Ultraschalluntersuchung konnte ein Entzündungsgeschehen und mittels MRT eine differenzialdiagnostisch diskutierte Läsion der Nervenwurzel L5 ausgeschlossen werden. Auf Basis der Befunde wurden eine Therapie mit Pregabalin (75mg; 1–0-1), lokalen Lidocain-Pflastern und eine Physiotherapie mit 2-Zellenbad eingeleitet, worunter sich eine leichte Besserung der Beschwerden ergab. In der folgenden neurografischen Verlaufskontrolle bestätigte sich ein Grenzbefund für eine (sehr geringe), nicht progrediente axonal-sensible Polyneuropathie, vereinbar mit einer Chemotherapie-induzierten Polyneuropathie; allerdings wurde die Symptomatik als untypisch eingeschätzt.

Im sechsten Jahr nahmen die Beschwerden wieder zu. Erstmals wurde ein deutlicher zeitlicher Zusammenhang zwischen den schubförmig auftretenden Schmerzen und den regelmäßig durchgeführten MRT-Untersuchungen durch die Patientin bemerkt. Eine Zunahme der Beschwerden trat regelhaft einige Tage nach intravenöser Gabe des hepatozytenspezifischen, linearen Gadolinium-Kontrastmittels (Gadoxetat) und unabhängig von den Zyklen der Systemtherapie und der Art der Medikation auf; die Symptome klangen dann langsam über mehrere Wochen, wenn auch unvollständig, wieder ab. Dabei standen nächtliche Bewegungsstörung, brennende Schmerzen der Fußrücken und Muskelkrämpfe und -faszikulationen im Vordergrund. Zeitgleich wurde der Verdacht auf einen Morbus Addison endokrinologisch ausgeschlossen, welcher bei dunkler Hautverfärbung der Hände, geäußert wurde.

Nach der Umstellung auf ein makrozyklisches Kontrastmittel sistierten alle Beschwerden vollständig und traten während der 10 folgenden Untersuchungen nicht mehr auf, was eine mögliche chronische Gadolinium-Toxizität nahelegt.

## Diskussion

Das Krankheitskonzept GDD ist hoch umstritten; Überlappungen mit Fibromyalgie oder ähnlichen Konditionen werden beschrieben. Wir berichten hier einen Fall, bei dem Beschwerden in zeitlichem Zusammenhang zu häufiger Applikation von linearem leberspezifischem Kontrastmittel (Gadoxetat) aufgetreten sind und nach Umstellung auf makrozyklische Präparate sistierten.

Semelka und Ramalho 5 schlagen für die Diagnose nach Ausschluss anderer Erklärungen vor, dass die Symptome nach Applikation des Kontrastmittels auftreten, in einem zeitlichen Zusammenhang stehen und zuvor nicht vorhanden sein dürfen. Zusätzlich sollen akute Schübe unter Gabe hochwirksamer Chelatoren induzierbar sein. Letzteres wurde in dem hier beschriebenen Fall nicht getestet. Ebenso wenig wurde der Versuch unternommen, Gadolinium in Gewebe oder Urin nachzuweisen, sodass trotz der engen zeitlichen Korrelation eine Unsicherheit ob der richtigen Einschätzung bleibt.

GDD wird gegenwärtig nicht mehr primär als toxischer Effekt des Gadoliniums, sondern als komplexe zytokinvermittelte Reaktion verstanden, die ein anderes Muster als bei nephrogener systemischer Fibrose oder asymptomatischen Gadoliniumablagerungen aufweist 6 . Ein mitochondrialer Schaden, wie auch die Wirkung auf Ionenkanäle wie TRPC6, der bei der Entstehung von Juckreiz eine Rolle spielt, sind zudem plausibel, gegenwärtig aber kaum erforscht 7 .

Besonders betroffen sind Frauen mitteleuropäischer Abstammung (wie die berichtete Patientin). Ein weiterer passender Risikofaktor sind allergische Konstellationen. Andere beschriebene Risikofaktoren sind autoimmune und genetische Konstellationen, besonders solche, die mit Eisenspeichererkrankungen einhergehen. Als Symptome werden Fatigue, „Brain Fog“, Schmerzen in Kopf, Muskulatur, Gelenken und Knochen beschrieben, brennende Schmerzen der Haut und Muskelfaszikulationen, gastrointestinale Symptome und Hautveränderungen. Damit bestehen klinische Ähnlichkeiten zu der akuten Kontrastmittelreaktion genauso wie zur nephrogenen systemischen Fibrose. Zumindest einige dieser Symptome waren in diesem Fall zu finden, auch wenn sie in Teilen auf die Systemtherapie zurückgeführt werden könnten.

Als ursächliche Therapie für das GDD werden Chelatoren eingesetzt und symptomatisch behandelt. Wichtigster Faktor ist allerdings die Vermeidung der Exposition mit gadolinium-haltigen Kontrastmitteln oder – wie in diesem Fall – der Wechsel des Kontrastmittels auf ein stabileres Präparat.

## Schlussfolgerung

Der vorgestellte Fall verdeutlicht, dass gadoliniumhaltige Kontrastmittel – trotz ihrer hohen diagnostischen Relevanz – bei wiederholter Anwendung mit der gebotenen Sorgfalt und unter kritischer Indikationsstellung appliziert werden sollten. Die hier geschilderte Symptomatik entwickelte sich über Jahre, wurde jedoch erst spät mit der Kontrastmittelgabe in Verbindung gebracht. Dies spricht für eine mögliche hohe Dunkelziffer milder, bislang unerkannter Verläufe, insbesondere bei komplex vorbehandelten Tumorpatienten. Vor der Gabe insbesondere linearer Präparate wie Gadoxetat sollten Patienten über die seltene Möglichkeit eines GDD informiert und bei wiederholter Anwendung gezielt zu etwaigen Beschwerden befragt werden.

Treten neuartige Sensibilitätsstörungen oder unspezifische Beschwerden in zeitlicher Nähe zur Kontrastmittelgabe auf, sollten diese ernst genommen, systematisch dokumentiert und differenzialdiagnostisch abgeklärt werden. Die Sensibilisierung der Radiologie für das noch wenig verstandene Krankheitsbild ist dabei essenziell – nicht nur zur frühzeitigen Identifikation potenzieller GDD-Fälle, sondern auch, um das Vertrauen betroffener Patienten zu erhalten. Letztlich wird sich die Existenz und Häufigkeit des GDD nur durch kontinuierliche ärztliche Wachsamkeit, offene Kommunikation und wissenschaftlich fundierte Fallanalysen verlässlich beurteilen lassen.
